# Evaluation of diffuse reflectance spectroscopy for predicting age, species, and cuticular resistance of *Anopheles gambiae s.l* under laboratory conditions

**DOI:** 10.1038/s41598-023-45696-x

**Published:** 2023-10-28

**Authors:** Mauro Pazmiño-Betancourth, Victor Ochoa-Gutiérrez, Heather M. Ferguson, Mario González-Jiménez, Klaas Wynne, Francesco Baldini, David Childs

**Affiliations:** 1 School of Engineering, University of Glasgow, Glasgow, G12 8QQ UK; 2 School of Biodiversity, One Health & Veterinary Medicine, University of Glasgow, Glasgow, G12 8QQ UK; 3 School of Physics and Astronomy, University of Glasgow, Glasgow, G12 8QQ UK; 4 School of Chemistry, University of Glasgow, Glasgow, G12 8QQ UK

**Keywords:** Infrared spectroscopy, Entomology, Machine learning

## Abstract

Mid-infrared spectroscopy (MIRS) combined with machine learning analysis has shown potential for quick and efficient identification of mosquito species and age groups. However, current technology to collect spectra is destructive to the sample and does not allow targeting specific tissues of the mosquito, limiting the identification of other important biological traits such as insecticide resistance. Here, we assessed the use of a non-destructive approach of MIRS for vector surveillance, micro diffuse reflectance spectroscopy (µDRIFT) using mosquito legs to identify species, age and cuticular insecticide resistance within the *Anopheles gambiae* s.l. complex. These mosquitoes are the major vectors of malaria in Africa and the focus on surveillance in malaria control programs. Legs required significantly less scanning time and showed more spectral consistence compared to other mosquito tissues. Machine learning models were able to identify *An. gambiae* and *An. coluzzii* with an accuracy of 0.73, two ages groups (3 and 10 days old) with 0.77 accuracy and we obtained accuracy of 0.75 when identifying cuticular insecticide resistance. Our results highlight the potential of different mosquito tissues and µDRIFT as tools for biological trait identification on mosquitoes that transmit malaria. These results can guide new ways of identifying mosquito traits which can help the creation of innovative surveillance programs by adapting new technology into mosquito surveillance and control tools.

## Introduction

Malaria is a major threat to global public health with an estimated of 247 million cases and 619,000 deaths in 2022^1^ with the majority of them reported in Sub-Saharan Africa. The parasite is transmitted by mosquitoes of the genus *Anopheles*, with the four primary vector species in Africa being *An. gambiae*, *An. coluzzii, An. arabiensis* and *An. funestus*^2,3^. Vector control programs are a key component of malaria management^4–6^ and vector surveillance is an important tool to evaluate and monitor the impact of those programmes^7,8^.

Vector surveillance faces a variety of challenges. The identification of species is challenging since the main malaria vectors in Sub-Sahara Africa occur as a species complex whose members are morphologically identical. Moreover, important features for species identification (legs, wings, scales) may be damaged by collection methods (e.g. CDC traps) and, in addition a high degree of expertise is required to identify samples at the species level^9^. Molecular techniques (PCR, DNA sequencing^10,11^ and LAMP^12^) are used to identify these vectors but the costs of reagents and the requirement of specialised laboratory equipment make them expensive to implement and maintain for programmatic surveillance. Additionally, age-grading mosquitoes is also crucial to predicting malaria transmission and evaluating the impact of interventions, but different challenges hamper this key measurement in vector populations^13,14^. Vector age is epidemiologically important because mosquitoes need to survive > 10 days to allow the completion of the malaria parasite extrinsic incubation period^15^ and become infective^16^. A successful vector control intervention would shift an older mosquito population to a younger non-infectious population by changing its age structure^17^. Dissections are used to assess the reproductive status (parous and nulliparous) of female mosquitoes on which basis they can be classified into two broad categories, ‘young’ (nulliparous assumed to be < 4 days old^18^) and ‘old’ (parous assumed to be > 4 days old). This technique is difficult to perform, therefore, impractical on larger sample sizes. There are many other techniques that have been investigated for age grading including characterisation of cuticular hydrocarbons^19,20^, transcription gene profiles^21–23^ and mass spectroscopy^24^ but none of them have been implemented into routine surveillance due to the expense of reagents and equipment. Hence, there is a need to identify alternative techniques to overcome the challenges of species identification and age grading in mosquito populations.

The constant ongoing selective pressure from insecticides (mainly pyrethroids) used on Insecticide Treated Nets (ITNs) and Insecticide residual spray (IRS) has given rise to insecticide resistance in most African malaria vector populations^25,26^. The emergence of resistance has the potential to significantly erode the effectiveness of control^27^. Hence, detection and measurement of the degree of insecticide resistance in vector populations is also a crucial component of surveillance. Mosquitoes have developed a variety of mechanisms to survive insecticide exposure including target insensitivity by point mutations (for example *kdr*)^28^, metabolic detoxification (over-expression P450, esterase and glutathione S-transferase^29,30^), sequestration^31^ and cuticular resistance. In the latter, the leg cloak in the tarsus is modified by an increase of hydrocarbons in the epicuticle, reducing the insecticide uptake by thickening or changing the composition of the leg^30,32^. This has been identified using electron microscopy and differential quantification of cuticular hydrocarbons (CHCs) and chitin^32–34^. Point mutations can be detected using molecular methods (PCR) but more complex metabolic resistance mechanisms, a proper diagnosis at large scale is impossible. Lately, qPCR^35^ has been shown to be capable of diagnosing metabolic pyrethroid resistance but this requires expensive reagents which hinders implementation as part of routine surveillance in low income countries. The diversity of mechanisms of insecticide resistance makes its detection challenging.

In recent years, mid-infrared spectroscopy (MIRS) has been developed to identify biological traits in mosquitoes^36^. Spectroscopy uses light-matter interactions to generate information that can be used for identification. Molecular vibrations can absorb specific frequencies of the infrared light, which will be specific to the various functional groups inside the molecule; thus providing a fingerprint of the analyte^37–39^. MIRS has been used to identify biological traits in *Anopheles* to identify blood meals^40^ and predict the age and species of laboratory and field populations of *Anopheles* and *Aedes*^36,41–43^. In these studies, the most common method for collecting spectra is attenuated total reflection (ATR) spectroscopy. Although this has shown to be a is a robust technique for acquiring MIRS from some insects (e.g. flesh flies^44^), a limitation is that the sample needs to be pressed against the ATR crystal for maximum contact which renders the sample unavailable for some analysis. Moreover, small mosquito tissues (wings and legs) cannot be used because of their small size, limiting the characterisation of different parts that might be informative for specific traits (e.g., the cuticular thickening associated with resistance in mosquito legs). The potential application of MIRS for wide-scale vector surveillance could be improved through use of alternative non-destructive approaches to signal acquisition. One possibility is the use of diffuse reflectance which allows measurement of the flux per wavelength of light reflected in a scattered manner from a sample^45^. This is achieved by focusing the infrared beam using an ellipsoidal or aspherical mirror and collecting the scattered light using another ellipsoidal mirror. Diffuse reflectance spectroscopy involves shining a beam of infrared light onto a sample, with part of the light being reflected from the surface, and the other part entering the sample by refraction. The infrared radiation will be absorbed at sample specific wavelengths and the resulting scattered light is collected. The sample will absorb specific wavelengths depending on its composition^37,46^. A variation of this technique is micro-diffuse reflectance spectroscopy (µDRIFT). It incorporates a microscope into the system which allows the measurement of specific locations of the sample by reducing the field of view according to needs. This ability to see the specific sample and to obtain spectra from small areas makes it possible to analyse tissue-specific features that are prohibitive with other MIRS sample techniques such as transmission and ATR^47^. This could be a particular advantage for aspects of mosquito surveillance where the trait of interest (e.g., type of insecticide resistance) may be localized to specific body parts or tissues. This technique is well suited to understand tissue-specific signals. As it requires a microscope, which potentially increasing the processing time and cost, if proven successful its implementation will require additional developments to make it appropriate for use in the field.

Here, we identify the suitability of different mosquito tissues for analysis by diffuse reflectance micro-spectroscopy (µDRIFT) and evaluate the use of these MIRS spectra for the prediction of species, age, and the presence of cuticular resistance in African malaria vector species.

## Results

### Tissue suitability for MIRS µDRIFT

**Figure 1:**
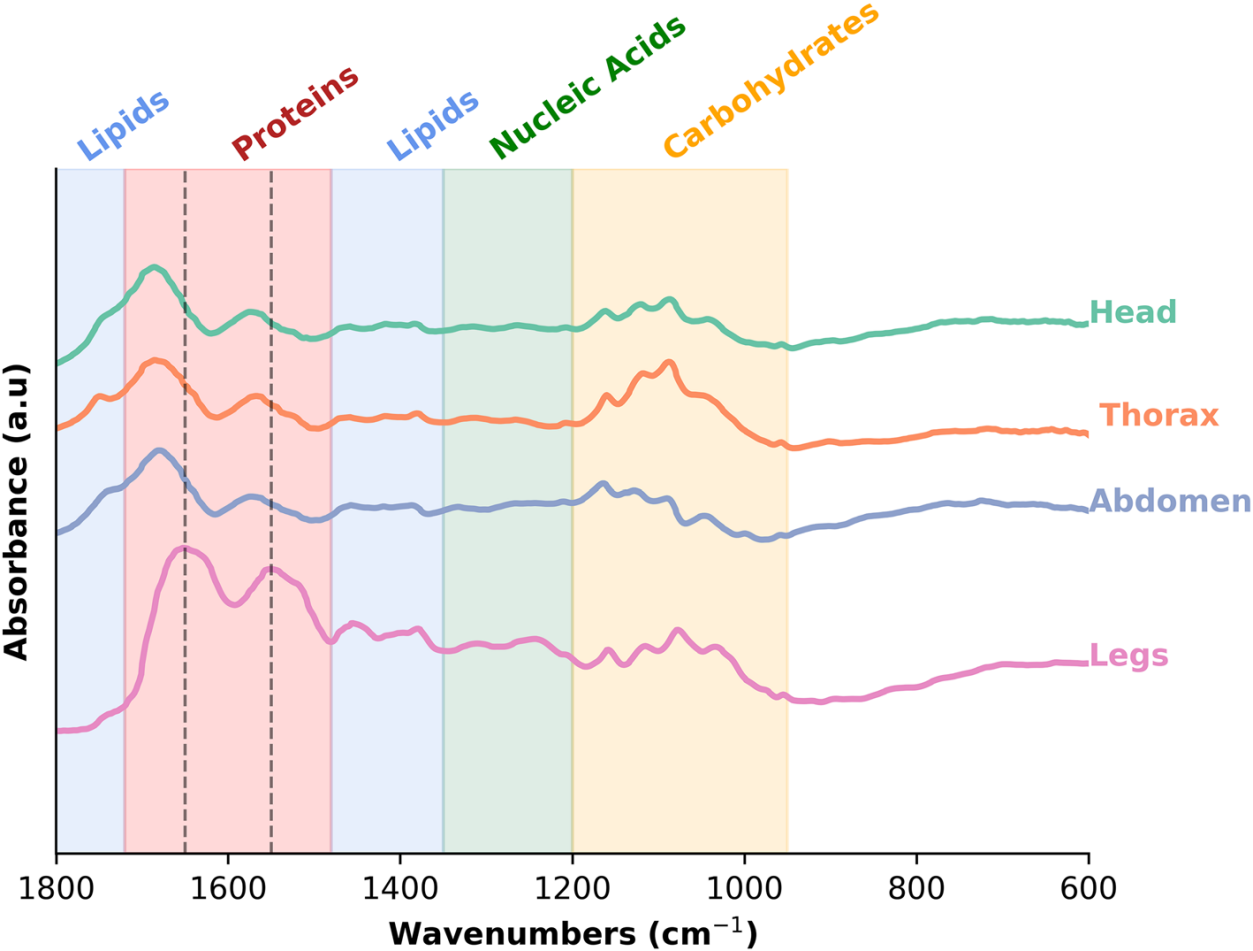
Spectrum of each type of mosquito tissue. Mean mid-infrared spectra of the head, thorax, abdomen, and leg from mosquito samples. Spectra baselines have been shifted for comparison. Coloured shadow regions show approximate biomolecular peak assignments from 1800 to 800 cm^-1^ region. Red dashed lines indicate amide I and amide II peaks at 1650 and 1550 cm^-1^ respectively.

First, we compared scanning procedure and collection of MIRS spectra using µDRIFT on different mosquito tissues. Scanning times varied depending on the mosquito body part. Thick tissues (head, thorax, abdomen) required up to 5 min (≈240 scans) of acquisition time to reduce noise. On the other hand, legs needed only 16 scans (20 s) to obtain a spectrum of sufficient quality (Fig. 1). Differences in spectra were most notable in the amide I and amide II bands where the peaks of both bands are shifted on thick samples. For example, the amide I band is shifted from 1650 cm^-1^ in the legs to 1685 cm^-1^ on the head, thorax, and abdomen. For amide II the shift was from 1550 cm^-1^ in the legs to 1575 cm^-1^ in thick tissues. Spectra obtained from different parts of the mosquito body were notably and consistently different. Subsequent analysis focused on analysis of leg samples given the considerably shorter acquisition time of signals from these tissues.

### Species and age classification

A total of 344 samples of 3 different replicates were used for age grading and species classification, which included two species *(An. coluzzii* and *An. gambiae)* and two age groups (3 and 10 days old) (Table 1).

**Table 1:** Species, strains, and age of the samples used for species and age classification using µDRIFT.

Species	Strain	# Samples	Age	# Cohorts
*An. gambiae*	Kisumu	64	3 days old	3
		105	10 days old	3
*An. coluzzii*	Ngousso	69	3 days old	3
		96	10 days old	3
Total		344		

### *Identification of* An. coluzzii *and* An. gambiae

**Figure 2:**
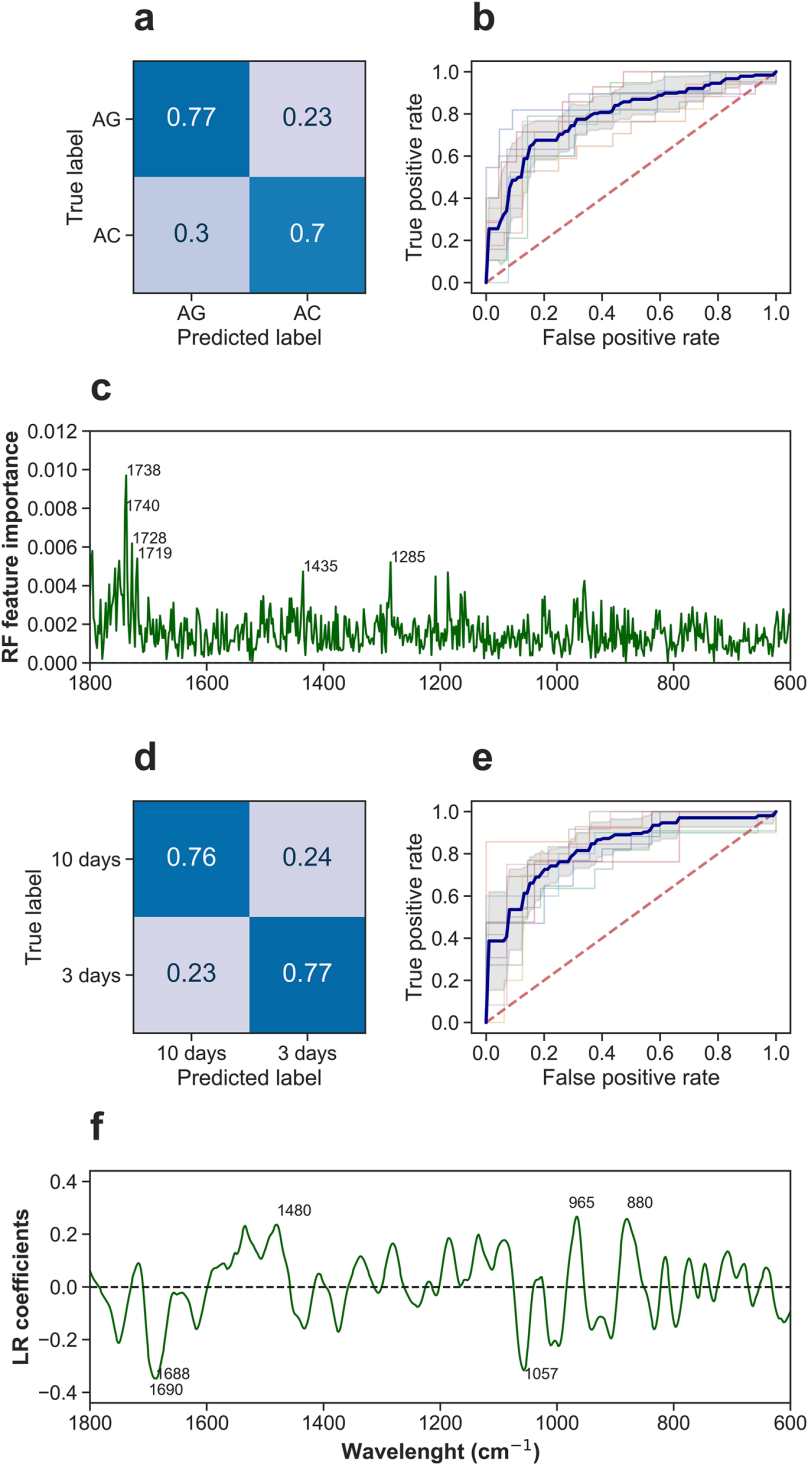
Model performance for and variable importance for species and age grading. Confusion matrix, receiver operating characteristic curve (ROC) values and variable importance for the prediction of *An. gambiae* (AG) and *An. coluzzii* (AC) (**a, b, c**) and age grading (**d, e, f**) using 10 models optimized and evaluated using nested cross-validation. Area Under the Curve (AUC) values are shown in Table 3 Variable importance of species and age prediction by mean decrease impurity for RF (species) **(c)** and model coefficients for LR (age) **(f)** show that the most important wavenumbers are located in the proteins (amide I) and chitin related regions. In panel (**b** and **e**) coloured lines represent the ROC curve of each model. Blue line is the mean ROC. Gray shaded area is the standard deviation. Red dashed line represents the ROC curve of a random classifier.

The MIRS spectra obtained from the legs were then analysed using machine learning algorithms. The final optimised model comprised of a total of 264 samples for training and was evaluated on a holdout set of 66 samples. Random Forest with multiplicative scatter correction (MSC) with second derivative Savitzky-Golay and a 9-window smoothing had the highest baseline accuracy (Supplementary material Figure S1). Nested cross-validation accuracy was 0.73 ± 0.6 and AU-ROC value of 0.77 ± 0.05 (Fig. 2a and b) while accuracy on the hold-out set was 0.71. The wavenumbers most heavily used by the algorithm to predict species were then analysed to understand the biochemical signature of this trait. Important features were located mainly in the 1750–1700 cm^−1^ region with some located in the 1400 and 1200 cm^−1^ regions (Fig. 2c). These wavenumbers are assigned to the C = O bond and C-CH_3_ bond which are related to wax, proteins and chitin and the C-O stretch related to chitin. Model evaluation by nested cross/validation and the hold out set suggested that MIRS spectra from legs collected with µDRIFT have a signal that can differentiate between these cryptic species.

#### Identification of 3 and 10 day old mosquitoes

For age classification, we used a logistic regression with smoothing using Savitzky-Golay with a window size of 9 points which obtained the best baseline performance when predicting the 3 and 10 day old age groups (Supplementary material Figure S2). Accuracy using nested cross-validation was 0.77 ± 0.06 which was lower compared to the hold out set accuracy (0.84) and AU-ROC value of 0.83 ± 0.06 (Fig. 2d and e). Analysis of the biochemical signature showed that the most important wavenumbers were located mainly in the Amide I and II region (1750–1550 cm^−1^), which related to proteins and chitin and in the C-CH_3_ and C–O stretch region (1400, 1000—900 cm^−1^) assigned to wax and proteins (Fig. 2f). This suggests that changes in the leg cuticle that are related to age in these two groups (3 and 10 days old) can be detected with µDRIFT and that our model can predict with similar accuracies both age groups.

### Identification of cuticular insecticide resistance

After the prediction of species and age was performed with supervised algorithms, the same approach was tested to analyses whether µDRIFT could differentiate the strain with cuticular insecticide resistance (Tiassale strain) from susceptible mosquitoes (Kisumu and Ngousso strains). For this, 291 samples of 4 different replicates for insecticide resistance classification (Tables 2, 3) were used.

**Table 2:** Species, strains, and age description of the samples use for insecticide resistance and strain identification using µDRIFT.

Species	Strain	# Samples	Age	Cohort	Group
*An. coluzzii*	Ngousso	30	1 day old	3	Susceptible
		30	2 days old	3	
		30	3 days old	3	
*An. gambiae*	Kisumu	30	1 day old	3	
		29	2 days old	3	
		29	3 days old	3	
*An. gambiae*	Tiassale	37	1 day old	4	Resistant
		39	2 days old	4	
		37	3 days old	4	
	Total	291

**Table 3:** Summary of the model evaluation metrics for each of the classification problems using nested cross-validation and hold-out set.

	Species		Age		Insecticide resistance	
	Nested CV	Hold out	Nested CV	Hold out	Nested CV	Hold out
Accuracy	0.73 ± 0.06	0.71	0.77 ± 0.06	0.84	0.75 ± 0.08	0.74
Sensitivity	0.71 ± 0.09	0.64	0.77 ± 0.13	0.78	0.65 ± 0.22	0.76
Specificity	0.76 ± 0.06	0.79	0.75 ± 0.08	0.89	0.87 ± 0.09	0.72
Precision	0.75 ± 0.10	0.75	0.74 ± 0.12	0.88	0.83 ± 0.14	0.73
AUC	0.77 ± 0.05	0.75	0.83 ± 0.06	0.92	0.83 ± 0.07	0.82

### Classification of “resistant group” (cuticular resistant strain) and “susceptible” (strains without cuticular resistant)

**Figure 3:**
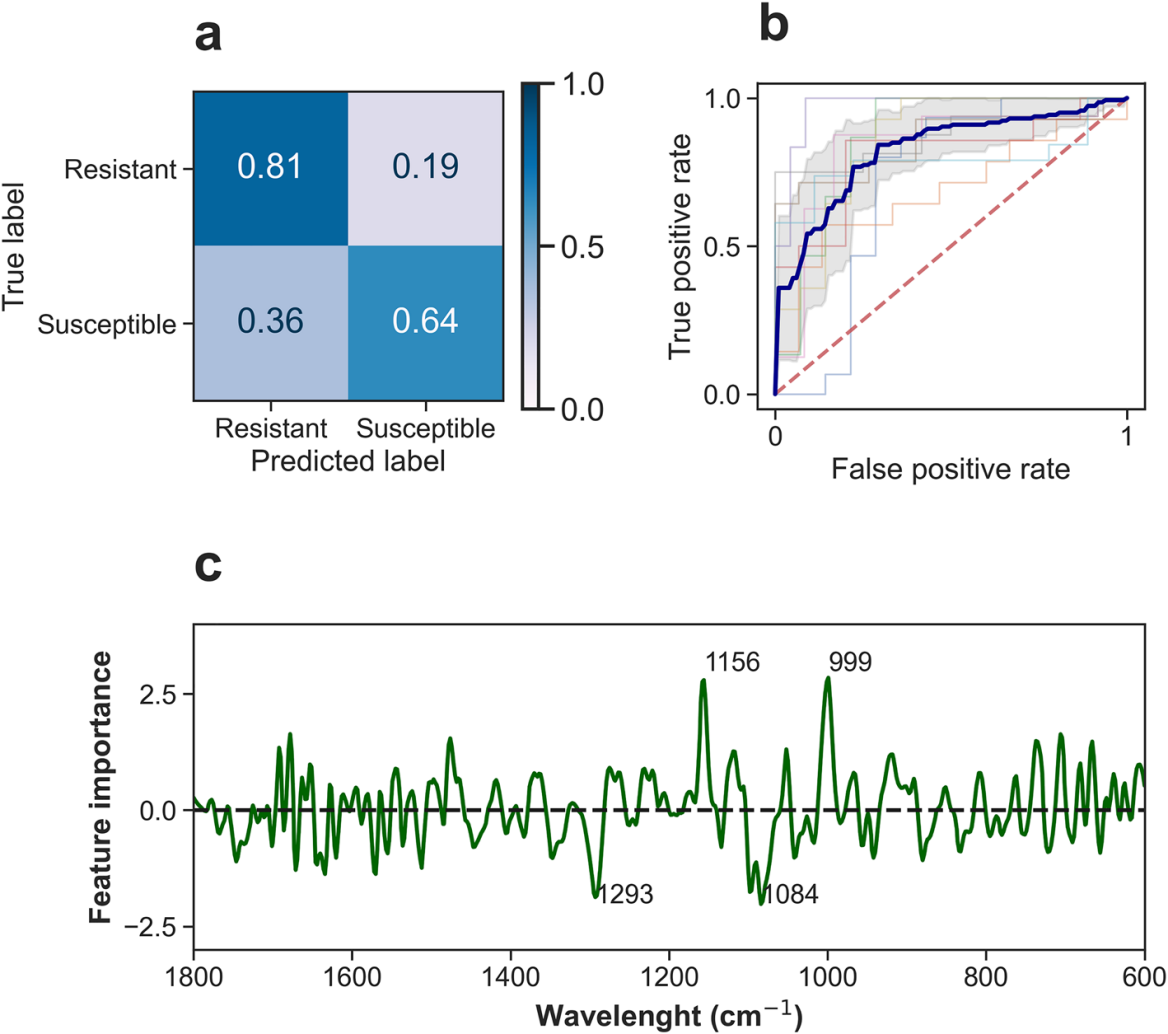
Model performance for insecticide resistance status. Confusion matrix (**a**) and Receiver operating characteristic (ROC) curves (**b**) for insecticide resistance status using 10 models optimized and evaluated using nested cross-validation. AUC values are shown in Table 3. (**c**) Variable importance by mean decrease impurity for RF. In panel (**b**), coloured lines represent the ROC curve of each model. Blue line is the mean ROC. Gray Shaded area is the standard deviation. Red dashed line represents the ROC curve of a random classifier.

Support vector machine (SVM) classifier showed the best performance when a first derivative Savitzky-Golay and smoothing with a window of 11 points was applied (Supplementary Material Figure S3). Model evaluation using nested cross-validation showed a mean accuracy of 0.75 ± 0.08 with a mean accuracy of 0.81 and 0.64 for resistant and susceptible classes, respectively, and AUC-ROC of 0.83 ± 0.07 (Fig. 3a and b). Accuracy using a hold-out set was 0.74. Most of the important features were in the C-O stretching (1084 cm^−1^) and C–O–C functional group (1154 cm^−1^) regions which are assigned to chitin (Fig. 3c). These results suggest that changes in the leg cuticle related to cuticular resistance can be detected with µDRIFT.

### Strain prediction

**Figure 4:**
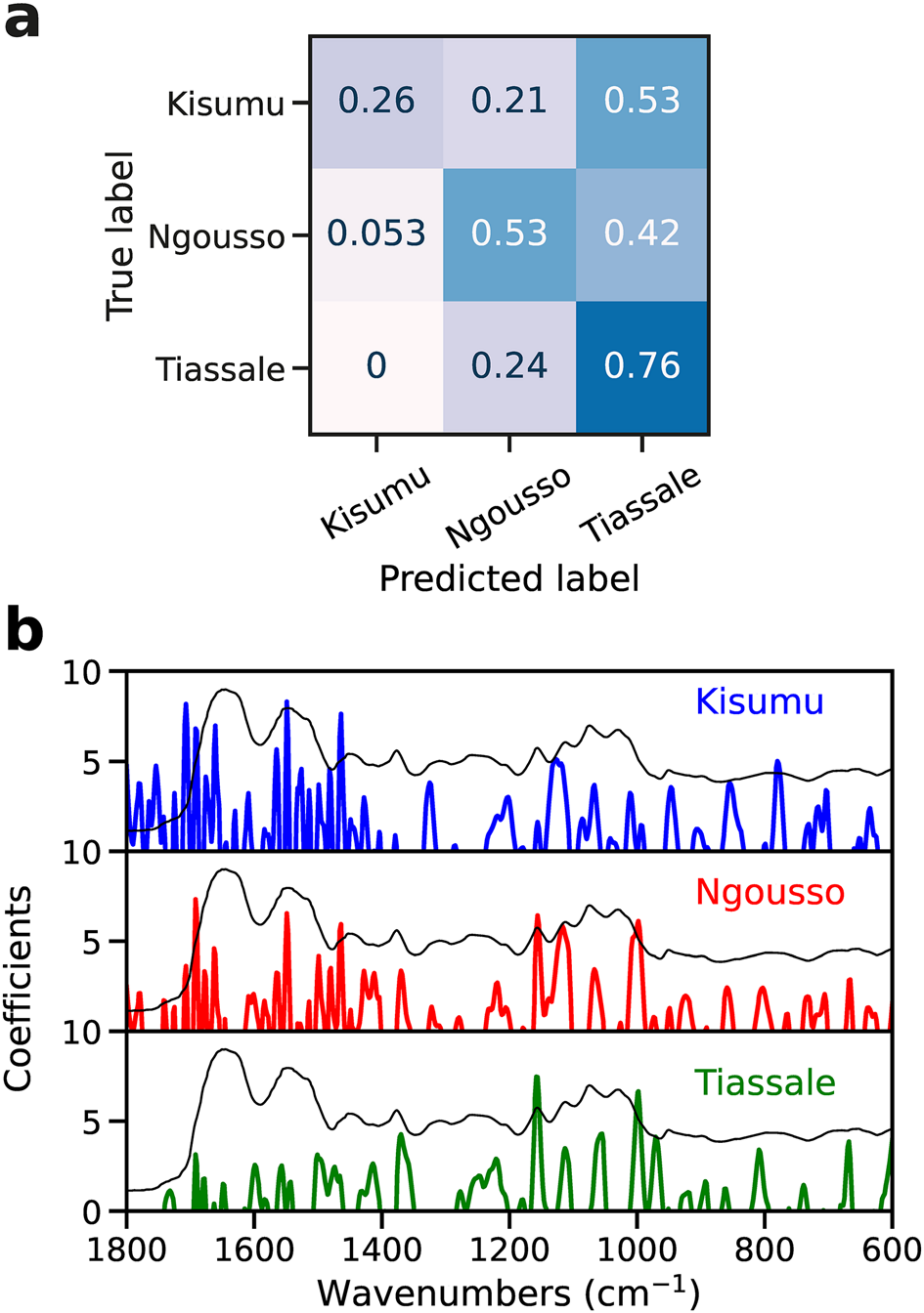
(**a**) Prediction of different strains. Normalised confusion matrices for SVM in validation set on multi-class strain classification. Each row represents instances of true class, while each column represents instances of the predicted class. (**b**) Prediction coefficients for each strain. Model coefficients of support vector machine classifier plotted against wavenumbers for each strain used. Multiclass strategy was ‘one vs the rest’ where each class is fitted to a classifier. Then on each classifier, the class is fitted against all other classes. Example spectrum in black was added for easy band identification.

To assess if MIRS was really predicting cuticular resistance, instead of some strain specific signal, we tested whether the MIRS signal could be used to differentiate between different strains. Our hypothesis was that, if MIRS was detecting a strain specific signal, we could differentiate all the strains; alternatively, if MIRS was detecting cuticular resistance only, the model could classify the resistance strain, but not the other two. Thus, a multi-class classification using the different strains with a SVM model was performed. Here the accuracy of the hold-out set was 0.63. The lowest prediction accuracy was for Kisumu (0.32), while Ngousso and Tiassale were had accuracies of 0.58 and 0.76 respectively (Fig. 4a). Prediction coefficients showed differences in weights for each strain. For Kisumu and Ngousso, most of the important wavenumbers were in the amide I, amide II and 1100 to 1200 cm^−1^ region. However, the prediction coefficients for Tiassale were mainly in the 1000 to 1200 cm^−1^ region (Fig. 4b). Most of the bands were related to chitin and wax. These results suggest that while insecticide susceptible strains could not be differentiated using µDRIFT, despite belonging to different species (*An. gambiae* and *An. coluzzii*), the cuticular resistant strain could still be accurately classified (which is a hybrid strain between *An. gambiae* and *An. coluzzii*); further suggesting that this resistance trait may be responsible for the biochemical signature learned by the algorithm.

## Discussion

This study showed the potential of µDRIFT as tool to identify biological traits in laboratory-reared *Anopheles* mosquitoes, including differentiating between *Anopheles gambiae* and *An. coluzzii* species, age classes and cuticular resistance. This technique allows for the collection of spectra from the head, thorax, abdomen, and legs individually without destroying the sample. Spectra from one leg taken from 344 mosquitoes analysed by random forest and logistic regression allowed us classify *An. coluzzii* and *An. gambiae* females with an overall accuracy of 0.77, but with relatively low accuracy for *An. coluzzii* (0.7). This is the first attempt to identify two species of the *An. gambiae* complex (*An. gambiae* and *An. coluzzii)* using µDRIFT. Important wavenumbers for model decision were mainly from the C = O stretch region (*circa* 1700 cm^−1^) and C-CH_3_ regions which are assigned to protein and wax^36,48^. Difference in the amount of cuticular hydrocarbons has been reported between these two species with *An. gambiae* showing greater amounts of hydrocarbons and a ticker cuticle compared to *An. coluzzii*^49,50^. Moreover, *An. coluzzii* shows increased survival on drier enviroments^51^ and greater desiccation tolerance^52^ which is linked to changes in the cuticle composition^50^ rather that cuticle thickness. This difference might be reflected on the wavenumbers used by the model to classify between the two species. µDRIFT has shown high classification accuracy between species of *Aedes* that are morphologically different (e.g., over 90% accuracy between *Ae. japonicus*, *Ae. triseriatus*, *Ae. albopictus* and *Ae. aegypti*)^53^. However, there are no comparable studies with µDRIFT using either MIRS or NIRS distinguishing these same *Anopheles* species, therefore, we cannot conclude that the low accuracy obtained here is due to the technique rather than the nature of the species. We hypothesise that some of the ecological differences between *An. coluzzii* and *An. gambiae* are associated with changes to the leg cuticle to allow reliable differentiation, however the classification power of our model may be limited by relatively low sample sizes.

Our model correctly predicts the age of 3 day and 10 day old mosquitoes with 0.84 with hold-out set. Although there are no other studies using µDRIFT applied to legs to predicting age, we obtained similar results to those reported for *Ae. aegypti* where females were correctly predicted into age categories of 2 and 10 days 81 to 88% accuracy respectively using mosquito abdomens and Partial Least Squares Discriminant Analysis (PLS-DA)^42^. González-Jiménez et. al.^36^ were able to predict *An. gambiae* into age groups 3, 9 and 11 with accuracies that ranged from 20—50%, and from 40—80% for *An. arabiensis*. The accuracy of predicting mosquito age into broad categories of "young" (< 7 days old) or "old" (7 + days) using NIRS has been reported to range from 65–97%^54–57^ . In NIRS analysis there is often variability in prediction accuracy between groups from 3 to 9 or 12 days, depending on the algorithm used^55,58^. The bands used by our model for age prediction were similar to those identified in two previous studies using MIRS^36,42^. Like these previous studies, we identified important bands for age prediction within the C-O stretching and amide I and II regions^48^ related to chitin, proteins and wax. It has been shown that gene expression changes with age in chitin bind cuticle proteins and genes associated with chitin metabolism in *Anopheles*^21,59^. These results provide further evidence that age affects the cuticle structure of the mosquito. These structural changes are associated with increases and decreases of cuticle proteins, and changes in the chitin metabolic process. Although these changes have not been fully characterised, they can be detected with MIRS.

To our knowledge, MIRS had never been tested for identification of cuticular insecticide resistance in mosquitoes. Cuticular resistance is characterised by a thickening of the leg cuticle by the increased content of chitin, proteins and cuticular hydrocarbons^32^, therefore can be potentially detected by MIRS. We found that the biochemical signature of resistance was associated with variation in the C-O stretching (940–1130 cm^−1^) and C–O–C stretch (1130–1170 cm^−1^) assigned to chitin and proteins. Mosquitoes with cuticular resistance have a thicker procuticle at the tarsus, an increment in cuticular hydrocarbons in the legs and a high content of chitin monomer D-glucosamine^32^. The differences may be due not only be in terms of chitin quantity, but also to qualitative differences arising from down-regulation of cuticular proteins (CPs) CPR8 and CPR120^32^. These differences may have been identified by the algorithm. The question remains as to whether this apparent difference can be pinpointed to cuticular resistance, or is a confounding effect of strain (e.g., only 1 strain had cuticular resistance). If there was a strain signal, we expected all strains to be classifiable. However, the model only identified the Tiassale strain, which was the only one with cuticular resistance. This further suggests that we are detecting cuticular resistance and not changes associated with strains. Further research is needed using multiple populations of the same species and strains with different degrees of cuticular resistance to assess how a model classifies them. Another option would be to measure the cuticle thickness (by electron microscope), chitin content from the legs and evaluate if they are correlated with the MIRS spectra of individual legs.

We evaluated our models using a holdout set and nested cross-validation to provide a more accurate representation of their performance. Evaluation using a holdout data set avoids any over or under-optimistic results; but we had over-optimistic accuracies in all our classification problems. This was especially high when predicting age, for which overall accuracy went from 0.84 using hold-out validation down to 0.77 with nested cross-validation. This phenomenon was less dramatic in models classifying species and cuticular resistance. These optimistically biased performance estimates from the ‘hold out’ approach can be linked to the small sample size used which is why nested cross-validation was also implemented. Nested cross-validation is known to give unbiased performance estimates regardless of sample size^60,61^. Also, an advantage of nested cross-validation approach is that it also gave ability to report metric values with a standard deviation. Future work should be performed with a larger sample size to confirm the accuracies reported here.

µDRIFT scanning time was different for tick tissues (head, thorax, and abdomen) and for legs. Long scanning times of up to 5 min were required for tick tissues. This scanning time is higher compared to scanning speeds reported in ATR spectroscopy and NIRS. However, the legs only required 16 scans with a resolution of 4 cm^−1^ which is less than 10 s. As mentioned in the methods section, a single mosquito needs approximately 35 s from removing the legs to collecting its spectrum. This scanning time is more efficient than parity dissection (8 min per mosquito Detinova method to 45 min Polovodova method^18^) or PCR to identify cryptic species. Moreover, scanning time can be reduced even further by collecting only the region from 1800 to 600 cm^−1^ to match NIRS scanning time of 3 to 5 s. The WHO insecticide susceptibility tests require 1 h of insecticide exposition plus 24 h to measure mortality. At 20–25 samples per tube with six replicates, it can process roughly 150 female mosquitoes per test. The number of mosquitoes tested can be increased but it will require increasing the number of test tubes and personnel while spectroscopy just needs one operator and the spectrometer. In terms of processing time, µDRIFT is at the same level of NIRS and MIRS using ATR (Supplementary Material Table S3). This technique can be used as an additional or complementary method to improve the understanding of the underlying mechanism of resistant.

Diffuse reflectance spectroscopy is very sensitive at changes in the path length, and one difficultly encountered here was positioning of mosquito legs as flat possible over the gold mirror to avoid distorted spectra from the samples. The leg joints can interfere and create a gap between the tissue and the gold mirror that generates changes in the spectrum; an issue previously raised in Sroute et al.^53^. We tried to minimise this problem by separating the femur from the rest of the leg and eliminating the joints to avoid mispositioning the sample. This process increased the overall processing time in the beginning, but it can be done easily with training. Future work should focus on the analysis of other species (e.g., *An. arabiensis* and *An. funestus*) to assess whether the low accuracy in species discrimination reported here is due to the close genetic relatedness of the species used here or is a general feature of the approach. Also, further study on mosquito groups expanded to encompass the differences in terms physiological status is needed to increase the robustness of the technique.

This study had several limitations. First, resistance or susceptibility was not validated by traditional techniques. Therefore, it is not certain that all individual tested were 100% resistant or susceptible. However, at the time of analysis, these strains were tested using WHO bioassays, and confirmed as ‘resistant’ or ’susceptible’ based on standard criteria^62^. Second, the decision of measuring only the legs for species identification and age grading was due to the longer scanning times required for thick tissues (head, thorax, abdomen) compared to the legs. The literature correlates chemical information with age in the head and thorax. However, legs have been also used for age relating studies^63,64^ and, also recent literature^53^ identified the legs as a useful target tissue for MIRS in species identification. In addition, cuticular resistance is known to be localised in the legs^30,32^. All these reasons made the case to focus on the legs for assessment of µDRIFT here in this study. However, the remaining tissue parts should be considered and explored in detail to take advantage of additional chemical information that may be present in the thorax and the head. Finally, the method still needs to be evaluated on semi field and field mosquitoes to assess the added benefit of the use of different tissues for prediction of biological traits using MIRS.

Diffuse reflectance spectroscopy offers a rapid method for the measurement of mid-infrared spectra of mosquitoes for species, age, and insecticide cuticular resistant classification. The benefit of using the legs is the ability to scan numerous samples in rapid succession by placing them next to one another, facilitating a swift scanning process. Moreover, the non-invasive nature of the process is an advantage compared to ATR spectroscopy, which leaves the remaining mosquito specimen available for further analysis (e.g., PCR or ELISA analysis for infection etc.). It allows the exploration of tissues such as wings and legs, which might serve to identify other biological traits, in mosquitoes and other arthropod disease vectors. Overall, the accuracy of µDRFIT was comparable to ATR in terms of age grading. Nevertheless, the added cost and the size of the microscope can be a limiting factor in terms of cost (*circa* $25.000 for the added microscope). However, new infrared microscopes with Quantum Cascade Lasers (QCLs) are becoming cheaper and smaller than current FTIR systems. The latter are high-powered tuneable mid-infrared lasers all the light to be focused down to the diffraction-limited focusing and so measures small tissue features. In the near future, such QCL systems will become easier to implement and test. Both NIRS and MIRS techniques are non-destructive, but NIRS have been used more extensively with moderate reported accuracy for age grading, species prediction. Diffuse reflection opens the door for non-invasive and targeted measurements that can expand vector surveillance tools. On top of that, new technologies (i.e., QCL based scanning systems) have shown the potential to overcome the limitations of diffuse reflection measurements. Currently, they have been used on human dermis for non-invasive glucose monitoring^65,66^. This technique is a promising approach despite current limitations, and it is worth exploring its strengths with further experiments.

## Materials and methods

### Mosquito strains and rearing

Experiments were conducted on two morphologically indistinguishable African malaria vector species: *Anopheles gambiae* (Kisumu and Tiassale strains) and *An. coluzzii* (Ngousso strain). The *Anopheles coluzzii* Ngousso strain from Yaoundé, Cameroon^67^ is susceptible to all insecticides. The *Anopheles gambiae* Tiassale strain from southern Cote d’Ivoire^68^ is resistant to permethrin and deltamethrin, organophosphates, carbamates and organochlorines, and is classified as having cuticular resistance^35,69^, and although it comprises mostly *An. gambiae*, it also contains some *An. coluzzii* hybrids. Resistance in this strain is conveyed by a variety of mutations (L1014F kdr and G119S Ace-1) and the up-regulation of P450^35^. *The Anopheles gambiae* Kisumu strain from Kenya^68^ is susceptible to all insecticides. The strains were reared under standard insectary conditions (26 ± 1 °C, 80% ± 10% humidity, 12 h light:12 h dark cycle) at the University of Glasgow, UK. Larvae were fed on Tetramin tropical flakes and Tetra Pond Pellets (Tetra Ltd., UK). Pupae were transferred into cages for adult emergence. Adult mosquitoes were fed ad libitum on 5% glucose. A cohort consisted of pupae collected and separated in cages for emergence, and then held for either 3 or 10 days post-emergence for age grading and species discrimination and 1, 2, and 3 days post-emergence for insecticide-resistant experiments prior to scanning. Approximately 20–30 adult females of each age and strain were collected per cohort. Each replicate was reared and collected at different time points. Three replicates were used for each species and strain except *An. gambiae* Tiassale, which needed four replicates to compensate for the larger sample size of the other strains, since it was the only resistant strain and for classification problems, groups with roughly the same sample sizes are needed.

### Sample processing

The protocol used to process mosquito samples prior to µDRIFT measurements is described in González-Jiménez et al.^36^ with minor modifications. Adult females were separated using a manual aspirator, transferred into holding cups, and killed by exposure to chloroform for 20 min. Dead mosquitoes were transferred into silica gel desiccant-filled tubes and stored at 4 °C. Samples were kept between 3–5 days to dry them completely prior to scanning.

### Diffuse reflectance spectroscopy/scanning

For tissue suitability, a pool of 30 samples was used. The head, thorax and legs of each mosquito were scanned with different acquisition times which ranged from 20 s (16 scans) up to 5 min (240 scans) Each spectrum was taken from the mid-infrared range from 600 to 4000 cm^−1^ at 4 cm^-1^ resolution. For analysis using the legs***,*** one hind leg (the femur) was scanned. Spectra were measured using a nitrogen-purged Bruker Vertex70 with a Hyperion 1000 microscope (Bruker Corporation, USA) using a 15 × reflective objective and liquid-nitrogen cooled mercury cadmium telluride (MCT) detector and Globar light source. Spectra were taken in the mid-infrared from 600 to 4000 cm^−1^ at 4 cm^-1^ resolution using 16 scans. Background measurements were taken every half hour while assessing the CO_2_ region.

### Data analysis

Import of individual files, assembling datasets, pre-processing and analysis of the spectra were performed using Python (Python 3.10.6) with in-house developed scripts. Before the analysis, the region from 1800 – 600 cm^-1^ was selected to reduce the number of features.

### Species prediction and age grading

Species prediction and age grading were based on the analysis of *An. gambiae* and *An. coluzzii* mosquitoes of ages 3 and 10 days old. First, the data set comprised of 3 cohorts. The classes were balanced and then the data was split into a training set (80%) and hold-out set (20%). The number of samples for each set are shown in Supplementary Table S1. Baseline performance of different machine learning algorithms and pre-processing methods was assessed using ten-fold stratified cross-validation with the training set (Supplementary Table S2). The algorithm and pre-processing combination with the highest baseline accuracy was then chosen for further optimisation using hyperparameter tuning. The final optimised model was then evaluated with the hold-out set. To overcome the limited sample size and to provide an unbiased estimation of performance, nested cross-validation was also implemented^60^. For this analysis, the whole data set was used. Nested cross-validation consists of two layers, the outer and the inner layer. In the outer layer, data was split into 90% for developing the model and 10% for validation. In the internal layer, the remaining 90% of the data was used for optimisation of the model by hyperparameter tuning using a three-fold cross-validation. The optimised model was then validated with 10% of the data which was split at the beginning. The whole process was repeated 10 times. Each time, a different 10% and 90% of the data set was selected for validation and model development, respectively. Mean accuracy, sensitivity, specificity, and Area under the curve (AUC-ROC) from the 10 optimized models is reported. Finally, the variable contribution was calculated using the coefficients when using logistic regression or support vector machine with a linear kernel. For random forest by Gini importance. All the training and evaluation was performed on Python using the scikit-learn library^70^.

### Prediction of cuticular resistance

Analysis of insecticide resistance was based on the comparison between the lines known to be susceptible (Kisumu, Ngousso) and known to be resistant (Tiassale). After balancing the classes, we performed a multiclass classification between the three strains using the hold-out method approach (number of samples for training and hold out set shown in Supplementary Table S1). The model used a one vs the rest approach which involves splitting the multi-class dataset into multiple binary classification problems. A binary classifier is then trained on each binary classification problem and predictions are made using the model that is the most confident. Then, another classification was performed with the strains grouped together into two classes: resistant and susceptible to insecticide. The dataset was balanced based on the new two classes before analysis. For this classification problem, the hold-out set approach and nested cross-validation were applied. Performance metrics (accuracy, sensitivity specificity and AUC) and variable contribution were reported as well.

### Supplementary Information

Supplementary Information.

## Data Availability

All relevant data have been deposited in the Enlighten repository, and it is available at 10.5525/gla.researchdata.1517. All code used for signal pre-processing and machine learning can be accessed at: https://github.com/maurocolapso/ML-DRIFT_Pazmino_et_al_2023.

## References

[1] Geneva: World Health Organization. *World malaria report 2022*. https://www.who.int/publications-detail-redirect/9789240040496.

[2] Coetzee M (2013). Anopheles coluzzii and Anopheles amharicus, new members of the Anopheles gambiae complex. Zootaxa.

[3] Sinka ME (2010). The dominant Anopheles vectors of human malaria in Africa, Europe and the Middle East: occurrence data, distribution maps and bionomic précis. Parasit. Vectors.

[4] Ogunah JA, Lalah JO, Schramm K-W (2020). Malaria vector control strategies. What is appropriate towards sustainable global eradication?. Sustain. Chem. Pharm..

[5] Benelli G, Beier JC (2017). Current vector control challenges in the fight against malaria. Acta Trop..

[6] Impoinvil DE (2007). Comparison of mosquito control programs in seven urban sites in Africa, the Middle East, and the Americas. Health Policy Amst. Neth..

[7] Russell TL (2020). Capacity of National Malaria Control Programmes to implement vector surveillance: A global analysis. Malar. J..

[8] World Health Organization. *Malaria surveillance, monitoring and evaluation: a reference manual*. (World Health Organization, 2018).

[9] Erlank E, Koekemoer LL, Coetzee M (2018). The importance of morphological identification of African anopheline mosquitoes (Diptera: Culicidae) for malaria control programmes. Malar. J..

[10] Chabi J (2019). Rapid high throughput SYBR green assay for identifying the malaria vectors Anopheles arabiensis, Anopheles coluzzii and Anopheles gambiae s.s. Giles. PLOS ONE.

[11] Bass C, Williamson MS, Wilding CS, Donnelly MJ, Field LM (2007). Identification of the main malaria vectors in the Anopheles gambiae species complex using a TaqMan real-time PCR assay. Malar. J..

[12] Bonizzoni M, Afrane Y, Yan G (2009). Loop-mediated isothermal amplification (LAMP) for rapid identification of anopheles gambiae and anopheles Arabiensis mosquitoes. Am. J. Trop. Med. Hyg..

[13] Muller GC (2017). The invasive shrub Prosopis juliflora enhances the malaria parasite transmission capacity of Anopheles mosquitoes: A habitat manipulation experiment. Malar. J..

[14] Emidi B, Kisinza WN, Mosha FW (2017). Impact of non-pyrethroid insecticide treated durable wall lining on age structure of malaria vectors in Muheza Tanzania. BMC Res. Notes.

[15] Beier JC (1998). Malaria parasite development in mosquitoes. Annu. Rev. Entomol..

[16] Brady OJ (2016). Vectorial capacity and vector control: reconsidering sensitivity to parameters for malaria elimination. Trans. R. Soc. Trop. Med. Hyg..

[17] White MT (2011). Modelling the impact of vector control interventions on Anopheles gambiae population dynamics. Parasit. Vectors.

[18] Hugo LE, Quick-miles S, Kay BH, Ryan PA (2008). Evaluations of mosquito age grading techniques based on morphological changes. J. Med. Entomol..

[19] Caputo B (2005). Identification and composition of cuticular hydrocarbons of the major Afrotropical malaria vector Anopheles gambiae s.s. (Diptera: Culicidae): Analysis of sexual dimorphism and age-related changes. J. Mass Spectrom..

[20] Suarez E (2011). Matrix-assisted laser desorption/ionization-mass spectrometry of cuticular lipid profiles can differentiate sex, age, and mating status of Anopheles gambiae mosquitoes. Anal. Chim. Acta.

[21] Wang M-H (2013). Gene expression-based biomarkers for anopheles Gambiae age grading. PLoS ONE.

[22] Cook PE (2007). Predicting the age of mosquitoes using transcriptional profiles. Nat. Protoc..

[23] Cook PE (2006). The use of transcriptional profiles to predict adult mosquito age under field conditions. Proc. Natl. Acad. Sci. U. S. A..

[24] Nabet C (2020). Prediction of malaria transmission drivers in Anopheles mosquitoes using artificial intelligence coupled to MALDI-TOF mass spectrometry. Sci. Rep..

[25] Camara S (2018). Mapping insecticide resistance in Anopheles gambiae (s.l.) from Côte d’Ivoire. Parasit. Vectors.

[26] Stica C (2019). Characterizing the molecular and metabolic mechanisms of insecticide resistance in Anopheles gambiae in Faranah. Guinea. Malar. J..

[27] Churcher, T. S., Lissenden, N., Griffin, J. T., Worrall, E. & Ranson, H. The impact of pyrethroid resistance on the efficacy and effectiveness of bednets for malaria control in Africa. *eLife* **5**, e16090 (2016). 10.7554/eLife.16090 PMC5025277 27547988

[28] Donnelly MJ, Isaacs AT, Weetman D (2016). Identification, validation, and application of molecular diagnostics for insecticide resistance in malaria vectors. Trends Parasitol..

[29] Liu N (2015). Insecticide resistance in mosquitoes: Impact, mechanisms, and research directions. Annu. Rev. Entomol..

[30] Balabanidou V, Grigoraki L, Vontas J (2018). Insect cuticle: A critical determinant of insecticide resistance. Current Opinion in Insect Science.

[31] Ingham VA (2020). A sensory appendage protein protects malaria vectors from pyrethroids. Nature.

[32] Balabanidou V (2019). Mosquitoes cloak their legs to resist insecticides. Proc. R. Soc. B Biol. Sci..

[33] Bass C, Jones CM (2016). Mosquitoes boost body armor to resist insecticide attack. Proc. Natl. Acad. Sci..

[34] Yahouédo GA (2017). Contributions of cuticle permeability and enzyme detoxification to pyrethroid resistance in the major malaria vector Anopheles gambiae. Sci. Rep..

[35] Mavridis K (2019). Rapid multiplex gene expression assays for monitoring metabolic resistance in the major malaria vector Anopheles gambiae 06 Biological Sciences 0604 Genetics. Parasit. Vectors.

[36] González Jiménez M (2019). Prediction of mosquito species and population age structure using mid-infrared spectroscopy and supervised machine learning [version 3; peer review: 2 approved]. Wellcome Open Res..

[37] Stuart, B. H. *Infrared Spectroscopy: Fundamentals and Applications*. *Methods* vol. 8 (2004).

[38] Larkin, P. J. *Infrared and Raman Spectroscopy: Principles and Spectral Interpretation*. *Infrared and Raman Spectroscopy: Principles and Spectral Interpretation* (2017).

[39] Tasumi, M. *Introduction to experimental infrared spectroscopy: Fundamentals and practical methods*. (Wyley and Sons, 2014).

[40] Mwanga EP (2019). Using mid-infrared spectroscopy and supervised machine-learning to identify vertebrate blood meals in the malaria vector. Anopheles arabiensis. Malar. J..

[41] Siria DJ (2022). Rapid age-grading and species identification of natural mosquitoes for malaria surveillance. Nat. Commun..

[42] Khoshmanesh A (2017). Screening of Wolbachia endosymbiont infection in Aedes Aegypti mosquitoes using attenuated total reflection mid-infrared spectroscopy. Anal. Chem..

[43] Santos MCD (2022). Infrared spectroscopy (NIRS and ATR-FTIR) together with multivariate classification for non-destructive differentiation between female mosquitoes of Aedes aegypti recently infected with dengue vs uninfected females. Acta Trop..

[44] Barbosa TM (2018). A novel use of infra-red spectroscopy (NIRS and ATR-FTIR) coupled with variable selection algorithms for the identification of insect species (Diptera: Sarcophagidae) of medico-legal relevance. Acta Trop..

[45] Workman, J. & Springsteen, A. *Applied Spectroscopy; A compact reference for practitioners*. (Academic Press, 1998). 10.1016/B978-0-12-764070-9.X5000-8.

[46] Mirabella, F. M. *Modern techniques in applied molecular spectroscopy*. (Wiley, 1998).

[47] Wihlborg, W. T., Reffner, J. A., Strand, S. W. & Wasacz, F. M. Reflection Spectroscopy With The FT-IR Microscope. in *7th Intl Conf on Fourier Transform Spectroscopy* vol. 1145 305 (SPIE, 1989).

[48] Machovič V (2017). Analysis of European Honeybee (Apis Mellifera) wings using ATR-FTIR and Raman spectroscopy: A pilot study. Sci. Agric. Bohem..

[49] Hidalgo, K. *et al.* Distinct physiological, biochemical and morphometric adjustments in the malaria vectors Anopheles gambiae and A. coluzzii as means to survive dry season conditions in Burkina Faso. *J. Exp. Biol.* **221**, jeb174433 (2018). 10.1242/jeb.174433 29378815

[50] Reidenbach KR (2014). Cuticular differences associated with aridity acclimation in African malaria vectors carrying alternative arrangements of inversion 2La. Parasit. Vectors.

[51] Yaro AS (2012). Dry season reproductive depression of Anopheles gambiae in the Sahel. J. Insect Physiol..

[52] Arcaz AC (2016). Desiccation tolerance in Anopheles coluzzii: the effects of spiracle size and cuticular hydrocarbons. J. Exp. Biol..

[53] Sroute L, Byrd BD, Huffman SW (2020). Classification of mosquitoes with infrared spectroscopy and partial least squares-discriminant analysis. Appl. Spectrosc..

[54] Sikulu M (2011). Evaluating RNAlater® as a preservative for using near-infrared spectroscopy to predict Anopheles gambiae age and species. Malar. J..

[55] Lambert B (2018). Monitoring the age of mosquito populations using near-infrared spectroscopy. Sci. Rep..

[56] Sikulu MT (2014). Non-destructive near infrared spectroscopy for simultaneous prediction of age and species of two major african malaria vectors: An Gambiae and an. Arabiensis. . NIR News.

[57] Sikulu M (2010). Near-infrared spectroscopy as a complementary age grading and species identification tool for African malaria vectors. Parasit. Vectors.

[58] Krajacich BJ (2017). Analysis of near infrared spectra for age-grading of wild populations of Anopheles gambiae. Parasit. Vectors.

[59] Wang MH (2010). Genome-wide patterns of gene expression during aging in the African malaria vector Anopheles gambiae. PLoS ONE.

[60] Vabalas A, Gowen E, Poliakoff E, Casson AJ (2019). Machine learning algorithm validation with a limited sample size. PLoS ONE.

[61] Varma S, Simon R (2006). Bias in error estimation when using cross-validation for model selection. BMC Bioinformatics.

[62] World Health Organization. *Test procedures for insecticide resistance monitoring in malaria vector mosquitoes*. (World Health Organization, 2016).

[63] Hugo LE, Kay BH, Eaglesham GK, Holling N, Ryan PA (2006). Investigation of cuticular hydrocarbons for determining the age and survivorship of Australasian mosquitoes. Am. J. Trop. Med. Hyg..

[64] Desena ML (1999). Potential for aging female Aedes aegypti (Diptera: Culicidae) by gas chromatographic analysis of cuticular hydrocarbons, including a field evaluation. J. Med. Entomol..

[65] Werth A, Liakat S, Dong A, Woods CM, Gmachl CF (2018). Implementation of an integrating sphere for the enhancement of noninvasive glucose detection using quantum cascade laser spectroscopy. Appl. Phys. B Lasers Opt..

[66] Isensee K, Kröger-Lui N, Petrich W (2018). Biomedical applications of mid-infrared quantum cascade lasers – a review. Analyst.

[67] Harris C (2010). Polymorphisms in Anopheles gambiae immune genes associated with natural resistance to plasmodium falciparum. PLoS Pathog..

[68] Williams, J. *et al.* Characterisation of Anopheles strains used for laboratory screening of new vector control products. *Parasit. Vectors* **12**, (2019). 10.1186/s13071-019-3774-3 PMC6833243 31690332

[69] Balabanidou V (2016). Cytochrome P450 associated with insecticide resistance catalyzes cuticular hydrocarbon production in Anopheles gambiae. Proc. Natl. Acad. Sci..

[70] Pedregosa F (2011). Scikit-learn: Machine learning in python. J. Mach. Learn. Res..

