# Supplementary material for: Evaluation of diffuse reflectance spectroscopy for predicting age, species, and cuticular resistance of Anopheles gambiae s.l under laboratory conditions
Source: Sci Rep. 2023 Oct 28;13:18499. doi: 10.1038/s41598-023-45696-x (PMC10613238; doi:10.1038/s41598-023-45696-x)
Supplement: Supplementary file 1 — Supplementary Information. [file 41598_2023_45696_MOESM1_ESM.docx]

**Evaluation of diffuse reflectance spectroscopy for predicting age, species, and cuticular resistance of *Anopheles gambiae s.l* under laboratory conditions**

Mauro Pazmiño Betancourth^1,2,*^, Victor Ochoa-Gutiérrez^1,3^, Heather M. Ferguson^2^, Mario Gonzalez-Jimenez^4^, Klaas Wynne ^4^, Francesco Baldini^2$^, David Childs^1$^

^1^ School of Engineering, University of Glasgow, Glasgow, G12 8QQ, UK.

^2^ School of Biodiversity, One Health & Veterinary Medicine, University of Glasgow, Glasgow, G12 8QQ, UK.

^3^ School of Physics and Astronomy, University of Glasgow, G12 8QQ, UK.

^4^ School of Chemistry, University of Glasgow, G12 8QQ, UK

[*mauro.pazminobetancourth@glasgow.ac.uk](mailto:*mauro.pazminobetancourth@glasgow.ac.uk)

^$^These authors equally supervised the work

**Supplementary Material**

**Supplementary tables**

**Table S1.** Number of samples used for training and test for each prediction.

| **Prediction** | **Total number of samples** | **Number of samples per class** | **Training set** | **Test set** |
| --- | --- | --- | --- | --- |
| Species | 330 | *An. gambaie* = 165  *An. coluzzii* = 165 | 264 | 66 |
| Age | 266 | 3 days old = 133  10 days old = 133 | 266 | 54 |
| Status | 286 | Susceptible = 143  Resistant = 143 | 228 | 58 |
| Strains | 273 | Ngousso = 90  Tiasale = 90  Kisumu = 93 | 219 | 54 |

**Table S2.** Pre-processing and machine learning algorithms used in the analysis

| **Pre-processing algorithms** |
| --- |
| Multiplicative Scattering correction (MSC) |
| Standard normal variate (SNV) |
| Robust normal variate (RNV) |
| Savitzky-Golay: 1^st^, 2^nd^ derivative, smoothing windows: 9, 11, 21 |
| **Machine learning algorithms** |
| Logistic regression (LR) |
| Random forest (RF) |
| Support vector machines (SVM) |
| Support Vector Machine with Stochastic Gradient Descent training (SGD) |

**Table S3:** Summary of the processing time of traditional methods for age grading (parity status), morphological identification, NIRS, MIRS using ATR and µDRIFT

| **Method** | **Sample preparation/ Scanning time per mosquito** | **Accuracy** | **Ref** |
| --- | --- | --- | --- |
| Parity dissection | 8 min Detinova  45 min Polovodova | Gold standard for parity status | ^1^ |
| Morphological identification | Not possible in cryptic species | – |  |
| NIRS | 30 seconds | High accuracy for species prediction and parity status in *Anopheles* | ^2–4^ |
| MIRS-ATR | Less than a minute | High accuracy for species and parity status in *Anopheles* | ^5,6^ |
| µDRIFT (legs) | 30 - 45 seconds | High accuracy for species prediction in *Aedes.*  Similar accuracies for age class prediction and species identification in *Anopheles* | ^7^, this study |

**Figure S1.** **Effect of pre-processing in model accuracy for species prediction** Overall prediction accuracy using the training set and ten-fold cross-validation for LR, RF, SGD and SVC models. Accuracy has been divided into scatter correction algorithm (no pre-processing (RAW), robust normal variate (RNV), standard normal variate (SNV), multiplicative scatter correction (MSC) and normalisation (NORM), filter window (9, 11 and 21 points) and derivative (no derivative, first and second derivative). Red dashed line indicates accuracy of a random classifier (accuracy of 0.5).

**Figure S2.** **Effect of pre-processing in model accuracy for age prediction** Overall prediction accuracy using the training set and ten-fold cross-validation for LR, RF, SGD and SVC models. Accuracy has been divided into scatter correction algorithm: no pre-processing (RAW), robust normal variate (RNV), standard normal variate (SNV), multiplicative scatter correction (MSC) and normalisation (NORM), filter window (9, 11 and 21 points) and derivative (no derivative, first and second derivative). Red dashed line indicates accuracy of a random classifier (accuracy of 0.5).

**Figure S3.** **Effect of pre-processing in model accuracy insecticide status identification** Overall accuracy using the training set and ten-fold cross-validation. Models used were: LR, RF, SGD, and SVC. Accuracy has been divided into scatter correction algorithm: no pre-processing (RAW), robust normal variate (RNV), standard normal variate (SNV), multiplicative scatter correction (MSC) and normalisation (NORM), filter window (9, 11 and 21 points) and derivative (no derivative, first and second derivative). Red dashed line indicates accuracy of a random classifier (accuracy of 0.5).

**Supplementary references**

1. Hugo, L. E., Quick-miles, S., Kay, B. H. & Ryan, P. A. Evaluations of Mosquito Age Grading Techniques Based on Morphological Changes. *Journal of Medical Entomology* **45**, 353–369 (2008).

2. Milali, M. P. *et al.* Age grading An. gambiae and An. arabiensis using near infrared spectra and artificial neural networks. *PLoS ONE* **14**, e0209451 (2019).

3. Milali, M. P. *et al.* An autoencoder and artificial neural network-based method to estimate parity status of wild mosquitoes from near-infrared spectra. *PLoS ONE* **15**, e0234557 (2020).

4. Sikulu, M. T. Non-Destructive near Infrared Spectroscopy for Simultaneous Prediction of Age and Species of Two Major African Malaria Vectors: An. Gambiae and An. Arabiensis. *NIR news* **25**, 4–6 (2014).

5. González Jiménez, M. *et al.* Prediction of mosquito species and population age structure using mid-infrared spectroscopy and supervised machine learning [version 3; peer review: 2 approved]. *Wellcome Open Research* (2019) doi:10.12688/wellcomeopenres.15201.3.

6. Siria, D. J. *et al.* Rapid age-grading and species identification of natural mosquitoes for malaria surveillance. *Nature Communications* **13**, 1–9 (2022).

7. Sroute, L., Byrd, B. D. & Huffman, S. W. Classification of Mosquitoes with Infrared Spectroscopy and Partial Least Squares-Discriminant Analysis. *Applied Spectroscopy* **74**, 900–912 (2020).
